# A Community-Based Nutrition and Physical Activity Intervention for Children Who Are Overweight or Obese and Their Caregivers

**DOI:** 10.1155/2017/2746595

**Published:** 2017-10-08

**Authors:** Furong Xu, Stephanie Marchand, Celeste Corcoran, Heather DiBiasio, Rachel Clough, Christopher S. Dyer, Jennifer Nobles, Jade White, Mary L. Greaney, Geoffrey W. Greene

**Affiliations:** ^1^Department of Kinesiology, University of Rhode Island, Independence Square II, Kingston, RI 02881, USA; ^2^KinderHealthRI, LLC, 10 High Street, Wakefield, RI 02879, USA; ^3^Coastal Medical Narragansett Bay Pediatrics, 65 Village Square Drive, South Kingstown, RI 02879, USA; ^4^Wakefield Pediatrics, LLC, 46 Holley Street, Wakefield, RI 02879, USA; ^5^Busy Bodies Studio, 12 High Street, South Kingstown, RI 02879, USA; ^6^Department of Nutrition and Food Sciences, University of Rhode Island, Fogarty Hall, Kingston, RI 02881, USA

## Abstract

There is a need for efficacious interventions to reduce the prevalence of childhood obesity, and a limited body of research suggests that collaborative community-based programs designed for children and their caregivers may be effective in reducing obesity rates. This paper reports the results of a community-based obesity intervention, South County Food, Fitness and Fun (SCFFF), designed for preadolescent children who are overweight or obese and their caregivers. SCFFF was developed in response to community concerns. Families were referred to the program by their physician and participated in the program at no cost. The 16-week intervention includes weekly group nutrition and physical activity sessions. Analyses determined that 65 out of the 97 children who completed SCFFF provided 2-year follow-up data and had reduced BMI *z*-scores over 2 years following the intervention. These participants decreased their energy, fat, carbohydrate, saturated fat, and sodium intake and increased core body strength and endurance from baseline to the end of the intervention. SCFFF was effective in reducing relative weight and improving diet and core muscle strength and endurance in children who are overweight or obese.

## 1. Introduction

Obesity is a major health concern among children in the United States [1]. Recent data indicate that 17.9% of children aged 6–11 years have obesity (body mass index (BMI) greater than or equal to the 95th percentile for children of the same age) [2]. Childhood obesity is associated with a number of adverse health risks, including increased risk of coronary heart disease, dyslipidemia, insulin resistance, hypertension, and weight-related psychological stress [3, 4]. Obesity in childhood often persists into adulthood [1, 3]. In addition, it poses significant economic burdens: medical costs are 30% higher for children who have obesity than children who stay at a healthy weight [5, 6]. Despite efforts to reduce childhood obesity, the percentage of children who are overweight or obese is remaining high [7].

Childhood obesity is a result of chronic positive energy balance with intake exceeding expenditure required for growth, homeostatic maintenance, and physical activity [8]. Pediatric weight management is a complex issue as obesity is a multifactorial condition due to environmental, social, and behavioral factors [9, 10]. Spear and colleagues recommend a four-stage pediatric obesity treatment approach that begins with prevention efforts plus structural weight management (stages 1 and 2) provided by primary care practitioners. If these efforts are not successful in changing the direction of relative weight gain, the next stage is a comprehensive multidisciplinary intervention to modify the child's behaviors and the home environment (stage 3). If the multidisciplinary intervention is unsuccessful or serious comorbidities exist, stage 4 interventions that include strenuous diet restriction, drug, or surgical treatment are recommended [11]. Although severe dietary restrictions and surgical interventions can reduce obesity among older adolescents and adults, neither approach is recommended for preadolescent children due to their nutritional and physiological needs as well as the risk of eating disorders [11, 12]. For preadolescent children with obesity, the goal is to reduce the rate of weight gain to allow adolescent maturation and physical growth to normalize body weight. Given the prevalence of childhood obesity, there is a clear need for primary care stage 1 and 2 interventions, but there are limited models of successful community-based stage 3 interventions for preadolescents who are overweight or obese to prevent obesity in adolescence [11, 13].

Numerous interventions have focused on improving dietary intake and increasing physical activity to reduce obesity in children [3, 14–16]. However, many of these interventions are school-based and may not consider the home setting [17]. Parents and guardians (henceforth referred to as caregivers) play an important role in determining what foods are available for their children and shaping their eating and physical activity behaviors [18]. Additionally, because children model physical activity and nutrition behaviors of family members, it is important to include caregivers in intervention efforts [18]. As a result, obesity prevention programs that include caregivers have a greater impact than those that do not involve them [19]. Nonetheless, there is still limited understanding of the effectiveness of community-based interventions (stage 3) that include caregivers in reducing relative weight with follow-up exceeding one year.

Thus, the primary aim of this study was to assess the effects of a community-based comprehensive stage 3, multidisciplinary intervention on BMI *z*-scores and BMI in children who are overweight or obese aged 6–10 years two years following the intervention. Secondary aims were to examine the effects of this intervention on children's dietary intake, physical fitness, and psychosocial functioning from baseline to postintervention.

## 2. Methods

### 2.1. Study Design and Participants

**Figure 1:**
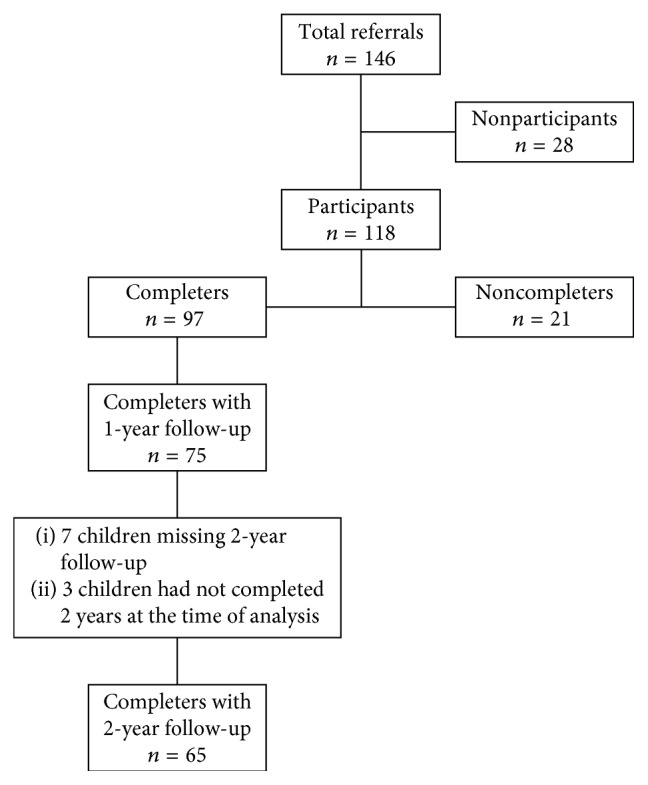
Study flowchart.

This was a single-group, longitudinal study examining the impact of South County Food, Fitness and Fun (SCFFF), a stage 3, multidisciplinary obesity intervention (described below). SCFFF was developed using social cognitive theory [20], and intervention activities were designed to facilitate and encourage children's healthy behaviors through positive interaction with their caregivers [20]. In total, 146 overweight (BMI greater than or equal to the 85th percentile but less than the 95th percentile for children of the same age, *n* = 21) or obese (BMI greater than or equal to the 95th percentile for children of the same age, *n* = 125) children between 6 and 10 years of age and their caregivers were recruited to participate in one of the fourteen 16-week SCFFF programs offered between February 2009 and December 2016 in Rhode Island [2]. Children and their caregivers were referred to SCFFF by their child's primary care physicians who determined that (1) they were free from medical conditions (e.g., severe heart disease) that limited eligibility, (2) they had not responded to stage 1 and stage 2 interventions in the primary care office, and (3) their family lacked resources for fee-based obesity prevention programs [11]. After attending an SCFFF orientation session, 118 children and their caregivers enrolled in the program, and 97 (82%) completed the intervention (see Figure 1 for study flowchart). Caregivers signed informed consent forms, and children signed assent forms. Caregivers also completed the Health Insurance Portability and Accountability Act (HIPAA) release forms that allowed referring physicians to release the anthropometric measures of participating children. This study was approved by the University of Rhode Island Institutional Review Board.

### 2.2. Intervention

SCFFF was created in response to local healthcare practitioners and community members identifying a need for effective obesity prevention programs for preadolescents [21, 22]. Pediatricians, dietitians, a child activity specialist, and nutrition and kinesiology faculty and students from a local university developed SCFFF using social cognitive theory [22]. In response to community concerns, SCFFF was provided at no cost to eligible families. Although initially funded by a local community hospital, the program is currently supported by grants from Blue Cross & Blue Shield of Rhode Island, fundraising activities, donations from the medical community, and volunteers from the university and community. Funding was secured to pay for program materials, the space, a pediatric dietitian, and a child activity specialist for 32 hours for each SCFFF cohort totaling 448 hours. Eight SCFFF board members (dietitians, pediatricians, university faculty, and community members) and over 50 university students volunteered about 3500 hours to SCFFF since 2009 with an average of 250 volunteered person-hours for each SCFFF cohort.

SCFFF took place at a community exercise and dance center for youth. The program included 16 weekly sessions (Table 1); each session lasted approximately 75 minutes and was attended by the child and at least one of their caregivers. At the first and last sessions, children completed physical assessments and parents completed self-administered surveys. The remaining 14 sessions focused on education and skill building and included nutrition and physical activity segments. Nutrition segments were led by a pediatric dietitian, and physical activity segments were led by a child activity specialist with 30 years of experience teaching children physical activity at the community level. The nutrition components of SCFFF encouraged substituting more healthful foods for foods with added sugar, processed foods, and fast foods without energy restriction. Physical activity was encouraged through games and fun activities that could be completed with equipment available at home. Caregivers and children met separately as groups for the nutrition and physical activity segments. Information was presented to caregivers in a round-table format that encouraged questions and sharing of ideas (e.g., strategies for overcoming barriers). Sessions for caregivers emphasized the importance of healthful eating for the whole family and of increasing the family's daily physical activity for health and well-being. Caregivers were provided handouts such as recipes and exercise tips. Nutrition and physical activity information was provided to children through interactive activities. At the end of each SCFFF session meeting, children and caregivers met for a 15 minutes of family physical activity. Student volunteers provided assistance at sessions under faculty supervision.

### 2.3. Outcome Measures

#### 2.3.1. Demographic and Anthropometric Measures

Caregivers reported the date of birth and sex of their participating children on a survey completed at the first session. The same pediatrician took anthropometric measurements on-site at both the first (week 1) and last (week 16) sessions. Children's height was measured to the nearest 0.25 inches with shoes removed using a Tanita stadiometer (model WB3000, Arlington Heights, Illinois), and weight in light clothing was measured to the nearest 0.1 lb on a balance-beam scale (model WB300731, Arlington Heights, Illinois). In addition, height, weight, and date of measurement were abstracted from the children's medical records at one and two years prior to SCFFF enrollment and for one and two years post intervention. Height and weight were used to determine BMI based on the Centers for Disease Control and Prevention growth chart [23]. BMI *z*-scores were computed using the Children's Hospital of Philadelphia Research Institute BMI *z*-score calculator for children [24]. BMI *z*-scores (deviation from normal BMI) were based on the participants' height, weight, sex, date of birth, and date of measurement. Change in BMI *z*-score was used as the study's primary outcome measure as they are age-related and are sensitive to changes in weight in children who are overweight or obese [25].

#### 2.3.2. Other Measures

Dietary intake was measured using the 152-item Youth Adolescent Food Frequency Questionnaire (YAQ, Harvard School of Public Health, Boston, Massachusetts, 1995) for the first nine SCFFF cohorts [26]. It was replaced by the 45-item Block Kids Food Screener (BKFS) for later cohorts based on participants' feedback about the lengthy questionnaire [27]. Both are valid and reliable methods of dietary assessment specifically designed for children and adolescents and widely used in research [28, 29]. Both YAQ and BKFS measure the same macro- and micronutrients that contribute to overall health (calories, protein, fat, carbohydrate, saturated fat, sodium, and added sugar) [26, 27]. YAQ data were analyzed by the Harvard School of Public Health following standard procedures, and data for the BKFS also were analyzed using standard procedures [29].

Physical fitness was measured using selected components of the FITNESSGRAM, a valid and reliable comprehensive fitness assessment in children aged 5–18 years [30]. The examined components included curl-ups, push-ups, trunk lifts, shoulder stretch, and back-saver sit and reach. Curl-ups, push-ups, and trunk lifts measure abdominal, trunk extensor, and upper body muscle strength and endurance while shoulder stretch and back-saver sit-and-reach components measure flexibility. All tests were implemented using existing protocols [30].

Psychosocial functioning was measured utilizing the 35-item Pediatric Symptom Checklist (PSC) for the initial eight cohorts, a valid and reliable approach to identifying psychosocial dysfunction [31]. The questionnaire uses a 3-point Likert scale (never = 0, sometimes = 1, and often = 2) and is summed to create an overall score of psychosocial impairment. In addition, three subscales were calculated: internalizing problems (5 items), externalizing problems (7 items), and attention problems (5 items) [32]. A summary score of 28 or higher or subscale scores of 5 or higher on internalizing, 7 or higher on externalizing, and 7 or higher on attention, respectively, suggest impairment in psychological functioning [32]. The PSC was replaced by the Pediatric Quality of Life Inventory (PedsQL 4.0) based on feedback from caregivers. The PedsQL is a 23-item survey and comprised four domains: physical functioning (8 items), emotional functioning (5 items), social functioning (5 items), and school functioning (5 items). It is a valid instrument to assess children's psychosocial health (mean of emotional, social, and school functioning subscales) and physical health (physical functioning subscale) through children's perception (self-report) and their parents' perception (parent report) [33]. Items were scored following standardized scoring procedures, with a higher score indicating a better health status [33].

### 2.4. Data Analysis

Program completers were defined as participants who attended more than half of offered sessions. Baseline differences between completers and noncompleters were analyzed using the Kruskal-Wallis or chi-square test. Pre- and postintervention differences in outcome measures for SCFFF program completers were determined by the paired *t*- or McNemar's test. Mixed models were used to analyze changes in BMI *z*-score and BMI from baseline to the final SCFFF session (week 16) and to 1 year and to 2 years following SCFFF participation in program completers. The sample size of noncompleters with 1-year (*n* = 3) or 2-year (*n* = 1) postdata did not allow for stable comparisons with completers; thus, all pre- and postintervention analyses include only completers. Missing follow-up data were imputed if data existed for adjacent points (4 values imputed). All analyses were performed using the SAS statistical analysis system (Version 9.3, SAS Institute, Cary, NC), and *p* < 0.05 was considered statistically significant.

## 3. Results

### 3.1. Characteristics of Study Participants

One hundred eighteen participants (50 boys, 68 girls), with a mean age of 8.4 ± 1.3 years and an average BMI *z*-score at baseline of 2.04 ± 0.46, enrolled in SCFFF, and 97 (82%) were classified as completers. BMI *z*-scores did not change in the two years prior to SCFFF participation (*β* = 0.021, 95% CI: −0.017, 0.058; *p*=0.277). Completers attended an average of 82% of sessions (SD = 17.6), and noncompleters attended an average of 30% of sessions (SD = 16.8). There were no differences in demographic or anthropometric characteristics between completers and noncompleters (Table 2).

### 3.2. Baseline to Postintervention Comparisons among Program Completers

Although BMI did not change from baseline to the last SCFFF session, BMI *z*-score decreased while height and weight increased over the course of the 16-week intervention (Table 3). Children decreased their intake of calories, carbohydrate, fat, saturated fat, and sodium over the intervention period when accessed via the YAQ or BKFS; changes in protein and added sugar intake were found in the BKFS assessment, not in the YAQ (Table 3). Participants (*n* = 97) increased their curl-up, push-up, and trunk lift scores, but shoulder stretch and sit-and-reach scores did not change (Table 3). Among participants with PSC data (*n* = 65), the mean summary PSC score and subscale scores decreased, but these changes were not statistically significant. There were five participants (8.3%) whose PSC scores suggested functional psychological impairment based on the standard scoring [31]. Among participants with PedsQL score (*n* = 28), the mean summary scores increased, but this was not statistically significant. The only change in subscale scores was the parental report of their child's emotional functioning, which increased significantly from 61.4 ± 17.2 at baseline to 68.9 ± 19.6 at postintervention (*β* = 7.90, 95% CI: 2.70, 13.11; *p*=0.004).

### 3.3. Changes in BMI *z*-Score and BMI in Program Completers with Follow-Up Data

**Figure 2:**
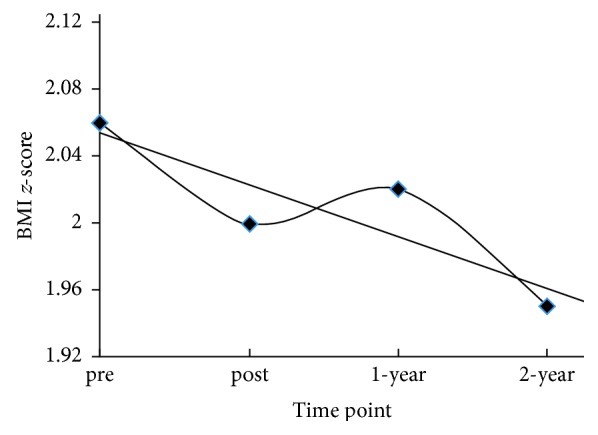
Overall change in BMI *z*-score in completers with 2-year follow-up data (*n* = 65). *Note.* Pre = baseline/premeasure; post = postmeasure; 1-year = 1 year following the intervention; 2-year = 2 years following the intervention.

The mean BMI *z*-score and mean BMI for program completers (*n* = 65) with 2-year follow-up data are presented in Figure 2 and Table 4. Among completers with 1-year follow-up (*n* = 75), BMI *z*-score decreased from 2.04 ± 0.44 at baseline to 2.01 ± 0.47 at one year, but this change did not reach significance (*β* = −0.017, 95% CI: −0.043, 0.010; *p*=0.216). Among completers with 2-year follow-up (*n* = 65; overweight = 10, obese = 55), BMI increased steadily (*β* = 0.87, 95% CI: 0.65, 1.08; *p* < 0.001), but BMI *z*-score decreased (*β* = −0.031, 95% CI: −0.054, −0.009; *p*=0.007) throughout the two-year period.

## 4. Discussion

This study found that SCFFF, a 16-week community-based obesity prevention program designed for children and their caregivers, was associated with a reduction in BMI *z*-score and energy intake. Analysis also determined that core body strength and endurance increased among SCFFF completers. The decrease in BMI *z*-score during the two years following the program indicates that the intervention effect was sustained for two years. This downward trajectory of BMI *z*-scores is likely related to caregivers' support and positive interaction with their child, which suggests that community-based interventions that include caregivers are a successful model for stage 3 multidisciplinary interventions. The long-term success of this type of program requires the collaborative community efforts of pediatricians, nutritionists, families, and others invested in the community [34].

Children were referred to SCFFF after stage 1 and stage 2 interventions were not successful, and they had demonstrated a persistently high relative weight for two years [35, 36]. The consistently high BMI *z*-score prior to participation in SCFFF may have been a precipitating factor in families' decision to participate, but motivations for enrolling in SCFFF were not assessed. Although the existing research indicates that not all parents perceive their children's weight status accurately [37–39], studies have determined that parents are motivated to enroll their children in intervention programs due to concerns about their children's weight especially if recommended by their primary care physician [11, 40, 41]. In our study, parental concern may have contributed to participants' relatively high completion rate (82%), which is consistent with Towey et al.'s study [42]. Completers had reduced BMI *z*-scores over the 16-week intervention and at 2-year follow-up with a downward trajectory. The effect size for change in BMI *z*-score (−0.15) is similar to that found in other family-based programs such as Fagg et al. and Trinh et al. [43, 44] but greater than that found by Kothandan [45].

The SCFFF nutrition education messages emphasized the need to increase nutrient-rich, non–energy dense foods such as fruits, vegetables, and whole grains and to limit consumption of energy-rich foods with added fats, salt, and sugars. Observed dietary changes indicate that children reduced fat and sodium intake, which suggests improvements in dietary quality. These dietary changes are consistent with those observed in other studies [46, 47]. It is important to note that SCFFF did not advocate energy restriction. The observed reduction in energy intake and BMI *z*-score was not associated with a reduction in weight or stature, both of which increased over the course of the program due to natural growth and development in youth at this age. Dietary assessment was initially measured by the YAQ and later switched to the BKFS to reduce participants' burden. Although both are valid instruments [28, 29], self-reported food frequency instruments are likely better for ranking than validity; thus, reported energy intake may not reflect true intake. The substantial differences in energy intake between these two instruments reinforce concerns about validity. Although caregivers were asked to complete the instruments with their children, it is possible that energy dense foods eaten away from home were underreported, although results of both the YAQ and BKFS are highly correlated with actual intake when compared to results of a 24-hour dietary recall [28, 48].

The SCFFF intervention was designed to improve children's physical fitness by having them develop skills to participate in fun physical activities that could be continued at home. The FITNESSGRAM assessment utilized assesses physical fitness, an indication of frequent participation in physical exercises with certain intensity level [49]. Program completers increased the number of curl-ups, trunk lifts, and push-ups they completed which reflect core strength and endurance. These results are similar to those found by Farris et al. who have examined the impact of a 12-week multidisciplinary intervention on the physical performance of children who are obese [50]. However, unlike Farris and colleagues' findings [50], participants in the present study did not show improvement in sit and reach (flexibility measures). This difference could be due to differences in the type of physical activity selected for the program. Farris and colleagues utilized traditional exercise approaches including warm-up, resistance exercises, aerobic activity, and stretching exercises in each session [50], whereas SCFFF was more focused on lifetime physical activities instead of addressing specific activities that improve flexibility. Though the measures assessed in this study do not directly assess physical activity, results suggest that making physical activity fun and facilitating family support may lead to improved physical fitness.

The slight but not statistically significant decrease in the PSC total scores and increase in the PedsQL total scores suggest that participating in SCFFF may have had a beneficial impact on children's psychological condition. However, only a few of children in this study (*n* = 5) were classified at risk using the PSC. Nonetheless, the positive changes in scores were consistent with previous research that determined that overall psychosocial health was influenced by internal factors of physical health [51]. The significant increase in children's emotional functioning as reported by parents on the PedsQL suggests that parents perceived their children were feeling better about themselves. It is worth noting that participants' mean PedsQL baseline psychosocial scores (<70, *n* = 28) were much lower than those of healthy children (>81, *n* = 5480) reported in other studies [52, 53]. The prevalence rate for psychological dysfunction is higher among children who are overweight or obese [54–56]. Further research is needed to assess the psychological effect of stage 3 intervention programs.

Given the importance of families in shaping dietary- and nutrition-related behaviors [18], the sustained participation of caregivers is an important study finding. The need to include caregivers in obesity intervention programs has been recognized as an important strategy to address childhood obesity, yet few programs actively engage caregivers and those generally have high rates of attrition [57, 58]. The 18% attrition rate in this study suggests that the intervention designed to be fun for children and engaging for caregivers was effective, although there is room for improvement.

Study strengths include the use of standardized and validated instruments and two-year follow-up. Nevertheless, the study is not without limitations. Dietary and physical activity behaviors and BMI of siblings and parents were not assessed. In addition, parents' facilitation of child and family activity outside of the intervention were not assessed. Parental income and education data were not collected; thus, comparisons with other studies are not possible. However, some degree of economic challenge can be assumed because children and their caregivers were referred to this no-cost program by their pediatrician who perceived that they did not have sufficient resources to pay for such a program. Participants were from a suburban area that is predominately white, which limits generalizability. Results cannot be applied to different populations, particularly minority and populations without regular primary care physicians. Moreover, this study employed a nonexperimental design with a single group and did not include a control group; thus, the anthropometric changes may be due to additional factors beyond the intervention.

## 5. Conclusion

Results of this study indicate that a community-based stage 3 intervention program for children and caretakers that focused on nutrition and physical activity can reduce relative weight in overweight and obese children. Future interventions should be tested using randomized controlled trials with more diverse sample and should assess caregiver and sibling behavior changes. Given the lifelong adverse health consequences of pediatric obesity, interventions that change the relative weight trajectory, such as the current study, should be widely implemented in community settings. Future studies need to address family involvement and collaboration within the community as it is crucial for the program effectiveness and long-term sustainability.

## Figures and Tables

**Table 1:** Description of the SCFFF program.

Audience	Nutrition segment	Physical activity segment
Child	Nutrition (30 minutes) (1) Games focusing on Food Guide Pyramid/Choose MyPlate food groups and serving sizes (topic of the week) (2) Food tasting games (3) Importance of fruits, vegetables, and whole grains (4) Awareness of foods high in added sugar, sodium, and fats (5) Snacks versus treats (6) Meals, fast foods, and beverages (7) Weekly Nutrition Challenge: making recipe with caregivers or arts and crafts related to topic of the week	Physical Activity (30 minutes of moderate to vigorous activity) (1) Description and demonstration of target activity (2) Weekly activity (rope jumping, ball playing, team games, yoga, hand-eye coordination) engages children fully (e.g., breathing quickens or sweating) for the entire 30 minutes with appropriate verbal cues to keep them on task or maintain pace (3) Weekly activity challenge

Caregivers	Nutrition (30 minutes) (1) Review the previous week's Nutrition Challenge and recipe tasting (2) Food Guide Pyramid/Choose MyPlate food groups and serving sizes (3) Foods high in added sugar, fats, and sodium (4) Snacks versus treats (5) Meals, fast foods, and beverages (6) Changing eating behaviors as a family (7) Importance of fruits, vegetables, and whole grains (8) Challenges in changing a child's eating behavior (9) Caregivers as role models for healthful eating (10) Weekly Nutrition Challenge recipe	Physical Activity (30 minutes) (1) Review the previous week's physical activity challenge, address barriers, and suggest resolutions for being physically active (2) Types of physical activities and activity intensity levels (3) Importance of physical activity for health (4) Increasing physical activity in daily life using available neighborhood resources (e.g., bike paths) (5) Parents as role models for active living (6) Child's weekly physical activity challenge

Child and caregiver	—	Interactive physical activity (15 minutes) (1) Child teaches caregiver how to do activity (2) Joint activity based on targeted physical activity

*Note.* SCFFF = South County Food, Fitness and Fun.

**Table 2:** Comparison of the baseline information (preintervention) by participation status.

	Total *n* = 118	Completers *n* = 97	Noncompleters *n* = 21	*p* value
Age (years)	8.4 ± 1.3	8.5 ± 1.3	8.1 ± 1.4	0.204
Height (cm)	54.3 ± 3.9	137.9 ± 9.4	137.6 ± 10.9	0.368
Weight (kg)	105.9 ± 28.4	47.5 ± 12.2	47.6 ± 16.4	0.082
BMI (kg/m^2^)	24.9 ± 4.4	24.9 ± 4.0	25.1 ± 5.8	0.690
BMI *z*-score	2.04 ± 0.47	2.04 ± 0.46	2.07 ± 0.50	0.775
Sex, male [*n* (%)]	50 (42.4)	39 (40.2)	11 (52.4)	0.047^∗^

*Note.* Values are mean ± SD unless otherwise specified; *p* values were obtained by performing the Kruskal-Wallis test or chi-square test. ^∗^ *p* < 0.05.

**Table 3:** Comparison between post- and premeasures for SCFFF program completers (*n* = 97).

Variables	*n*	Pre	Post	Difference between post and pre (post − pre, 95% CI)	*p* value	Effect size
BMI (kg/m^2^)	97	24.9 ± 4.0	24.7 ± 3.7	−0.20 (−0.59 to 0.20)	0.117	−0.055
BMI *z*-score	97	2.04 ± 0.46	1.97 ± 0.45	−0.08 (−0.11 to −0.04)	<0.001^∗^	−0.150
Height (cm)	97	137.9 ± 9.4	140.0 ± 10.3	1.99 (1.30 to 2.70)	<0.001^∗^	0.585
Weight (kg)	97	47.5 ± 12.2	48.6 ± 12.4	1.11 (0.59 to 1.63)	<0.001^∗^	0.463
YAQ	65^a^					
Calories (kcal)		2105.6 ± 484.8	1888.5 ± 429.3	−217.15 (−316.49 to −117.81)	<0.001^∗^	−0.542
Protein (grams)		91.9 ± 21.8	89.4 ± 21.3	−2.50 (−7.03 to 2.02)	0.273	−0.137
Fat (grams)		70.2 ± 19.0	60.2 ± 14.9	−10.04 (−14.15 to −5.94)	<0.001^∗^	−0.606
Carbohydrate (grams)		283.4 ± 71.6	254.5 ± 66.6	−28.94 (−43.26 to −14.62)	<0.001^∗^	−0.501
Saturated fat (grams)		24.2 ± 7.0	20.8 ± 5.9	−3.35 (−4.81 to −1.89)	<0.001^∗^	−0.567
Sodium (mg)		2637.5 ± 679.5	2340.0 ± 523.2	−297.45 (−443.4 to −151.49)	<0.001^∗^	−0.505
Added sugar (tsp)		63.3 ± 26.9	57.3 ± 29.9	−5.99 (−12.43 to 0.44)	0.067	−0.231
BKFS	18^b^					
Calories (kcal)		1241.1 ± 448.2	910.3 ± 294.6	−330.78 (−565.21 to −96.35)	0.009^∗^	−0.705
Protein (grams)		57.4 ± 20.5	43.5 ± 13.7	−13.92 (−24.72 to −3.12)	0.015^∗^	−0.644
Fat (grams)		52.8 ± 22.6	35.0 ± 13.5	−17.74 (−28.61 to −6.86)	0.003^∗^	−0.815
Carbohydrate (grams)		138.6 ± 43.3	110.3 ± 33.1	−28.37 (−53.03 to −3.70)	0.027^∗^	−0.575
Saturated fat (grams)		18.4 ± 8.0	12.0 ± 4.5	−6.38 (−10.14 to −2.62)	0.002^∗^	−0.849
Sodium (mg)		2066.3 ± 774.4	1449.7 ± 442.3	−616.57 (−1027.47 to −205.68)	0.006^∗^	−0.750
Added sugar (tsp)		5.7 ± 3.2	3.2 ± 2.0	−2.57 (−4.37 to −0.76)	0.008^∗^	−0.711
PSC total score	65^c^	12.4 ± 8.3	11.2 ± 9.0	−1.20 (−2.67 to 0.27)	0.108	−0.202
PedsQL total (self)	28^d^	73.1 ± 18.0	74.6 ± 14.8	1.51 (−3.51 to 6.54)	0.542	0.09
Psychosocial health		67.6 ± 23.8	70.7 ± 17.2	3.10 (−3.21 to 9.40)	0.323	0.190
PedsQL total (parent)	28^e^	68.8 ± 13.2	72.8 ± 15.8	2.97 (−1.17 to 7.11)	0.152	0.20
Psychosocial health		66.1 ± 13.7	68.9 ± 18.3	2.86 (−1.96 to 7.68)	0.235	0.230
FITNESSGRAM	97					
Curl-up		9.67 ± 10.59	12.48 ± 8.96	2.72 (0.76 to 4.68)	0.007^∗^	0.280
Trunk lift (inches)		7.09 ± 2.90	8.23 ± 3.81	1.07 (0.43 to 1.72)	0.001^∗^	0.333
Push-up		5.22 ± 5.89	6.42 ± 5.67	1.30 (0.36 to 2.25)	0.015^∗^	0.279
Sit and reach (R) (inches)		−1.27 ± 3.22	−1.01 ± 2.93	0.27 (−0.21 to 0.74)	0.264	0.114
Sit and reach (L) (inches)		−1.21 ± 2.92	−1.23 ± 2.96	−0.02 (−0.53 to 0.49)	0.936	−0.008
Shoulder stretch (R) [*n* (%)]		56 (57.7)	63 (65.0)	61.8% (46.0 to 77.6)^f^	0.089^g^	0.618
Shoulder stretch (L) [*n* (%)]		44 (45.4)	50 (51.6)	72.0% (58.5 to 85.6)^f^	0.109^g^	0.720

*Note.* SCFFF = South County Food, Fitness and Fun. YAQ = Youth Adolescent Food Frequency Questionnaire. BKFS = Block Kids Food Screener. PSC = Pediatric Symptom Checklist. PedsQL = Pediatric Quality of Life Inventory. Values are mean ± SD unless otherwise specified; *p* values were obtained by performing the paired *t*-test except for the note. ^a^Data were collected from 1–9 cohorts. ^b^Data were collected from 10–14 cohorts. ^c^Data were collected from 1–8 cohorts. ^d, e^Data were collected from 9–14 cohorts. ^f^The risk difference = (proportion of post-Yes among all pre-Yes) – (proportion of post-Yes among all pre-No). ^g^ *p* value was calculated by McNemar's test (binary variables). ^∗^ *p* < 0.05.

**Table 4:** Relative weight for SCFFF completers with 2-year follow-up (*n* = 65).

Time points	*n*	BMI (kg/m^2^)	BMI *z*-score
Pre	65	25.0 ± 3.6	2.06 ± 0.42
Post	65	24.8 ± 3.3	2.00 ± 0.41
1-year	65	26.5 ± 4.3	2.02 ± 0.47
2-year	65	27.3 ± 4.5	1.95 ± 0.57

*Note.* Values are mean ± SD. SCFFF = South County Food, Fitness and Fun. 1-year = 1 year following the intervention; 2-year = 2 years following the intervention.
